# Use of Glucose Obtained from Biomass Waste for the Synthesis of Gluconic and Glucaric Acids: Their Production, Application, and Future Prospects

**DOI:** 10.3390/molecules30143012

**Published:** 2025-07-18

**Authors:** Mariya P. Shcherbakova-Sandu, Eugene P. Meshcheryakov, Semyon A. Gulevich, Ajay K. Kushwaha, Ritunesh Kumar, Akshay K. Sonwane, Sonali Samal, Irina A. Kurzina

**Affiliations:** 1 Faculty of Chemistry, National Research Tomsk State University, 36 Lenin Ave., 634050 Tomsk, Russia; meevgeni@mail.ru (E.P.M.); semen20200@gmail.com (S.A.G.); kurzina99@mail.ru (I.A.K.); 2 Department of Metallurgical Engineering and Materials Science, Indian Institute of Technology Indore, Indore 453552, India; akk@iiti.ac.in (A.K.K.); phd2201205005@iiti.ac.in (A.K.S.); phd2401105006@iiti.ac.in (S.S.); 3 Department of Mechanical Engineering, Indian Institute of Technology Indore, Indore 453552, India; ritunesh.kumar@iiti.ac.in

**Keywords:** glucose oxidation, gluconic acid, glucaric acid, heterogeneous catalysis, electrocatalysis, enzymatic catalysis, chemical synthesis, waste treatment

## Abstract

The demand for biomass has been growing in recent years for several reasons, related to environmental, economic, and social trends. In the context of global climate changes and the depletion of natural resources, the recycling of plant biomass waste is a promising strategy for sustainable development that contributes to minimizing waste, improving resource efficiency, and achieving the goal of creating a circular economy. One of the highly demanded products of agricultural waste recycling is glucose. Glucose is an important organic substrate that allows a number of value-added products to be obtained. In this review, we focused on the commercially significant products of glucose oxidation: gluconic and glucaric acids. This review summarized the latest available data on the scope of the application of each product as well as the methods of their production. The capabilities and limitations of currently used methods of synthesis were highlighted.

## 1. Introduction

**Figure 1:**
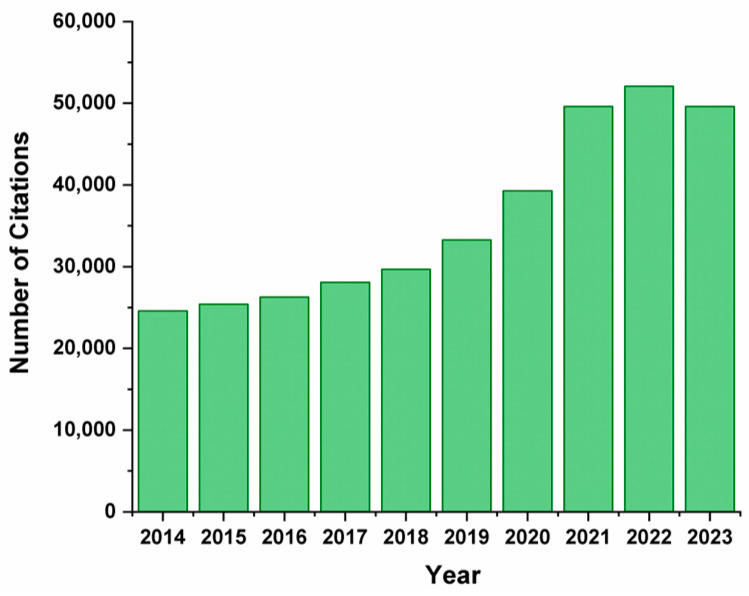
The number of citations on the recycling of plant-based waste into value-added products over the past decade.

In recent years, the demand for biomass has been growing worldwide due to the need to transition to a low-carbon bioeconomy. Renewable sources of raw materials are an alternative to fossil resources and have good prospects since they can be used for obtaining valuable products in the chemical industry. Over the last century, the world population has grown significantly; meanwhile, the global production and consumption of plant food have also increased [1]. Consequently, the generation of biomass waste, produced by the agro-industrial sector, increases annually, and its combustion leads to greenhouse gas emissions, which have serious consequences for the environment [2,3]. Biomass of plant origin represents a prospective renewable and sustainable source of energy, chemicals, and various carbon-containing materials [4,5,6,7]. The recycling of plant biomass waste is especially beneficial in terms of reducing economic expenditures, preventing waste burial, and, as a result, improving the environmental situation [8,9,10,11]. In addition, the use of renewable energy sources promotes energy security, reducing resource and energy expenditures and preventing environmental pollution [12,13]. Non-food plant crops, agricultural waste (seed and cereal husks, potato peelings, beet peels, sugar cane bagasse, corn husks, peanut shells, rice husks, wheat straw, etc.), and waste from the pulp and paper industry are of particular interest for recycling [1,14,15,16,17]. Over the past 10 years, the need for the recycling of plant waste has increased due to the growing demand for waste-free production and the development of new approaches to the utilization of raw materials (Figure 1).

**Figure 2:**
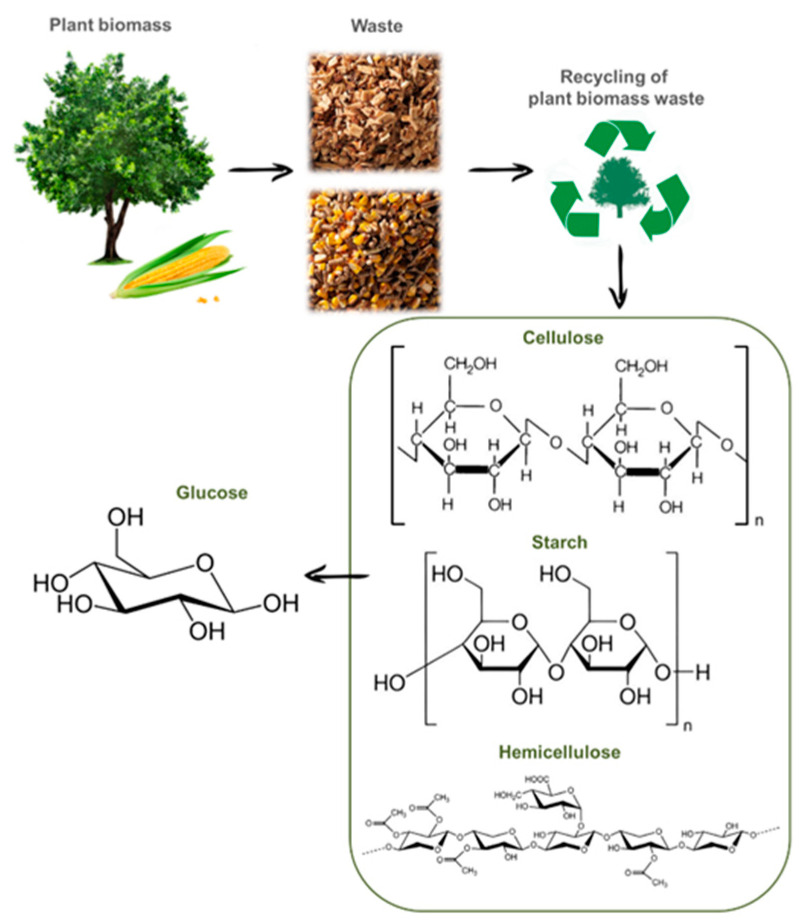
The scheme of the recycling of plant biomass into glucose.

The market for the use of chemicals of biological origin is also growing [2]. Particular attention is paid to strategies for the development of new technologies for the recycling of lignocellulose into high-value-added bio-products [14]. Renewable resources and by-products of plant-growing have great potential in terms of recycling for obtaining biofuels as well as products of special interest to industry [18,19,20]. Biomass and plant waste are rich in three main components: cellulose, hemicellulose, and lignin [21]. One of the most important products, obtained as a result of the hydrolysis of natural polysaccharides (cellulose and hemicellulose), is glucose (Figure 2) [22,23,24]. Potato peelings and corn husks are waste products of plant origin and contain starch, which is the raw material for obtaining glucose [24].

Glucose is a predecessor of a wide range of value-added products that are used in different spheres of the industrial sector [25]. The industrial glucose market was valued at 47.17 billion US dollars in 2024. The annual growth rate of the global glucose market is forecasted to increase by 4.8%, and it will reach nearly 80.4 billion US dollars by 2032 [26].

**Figure 3:**
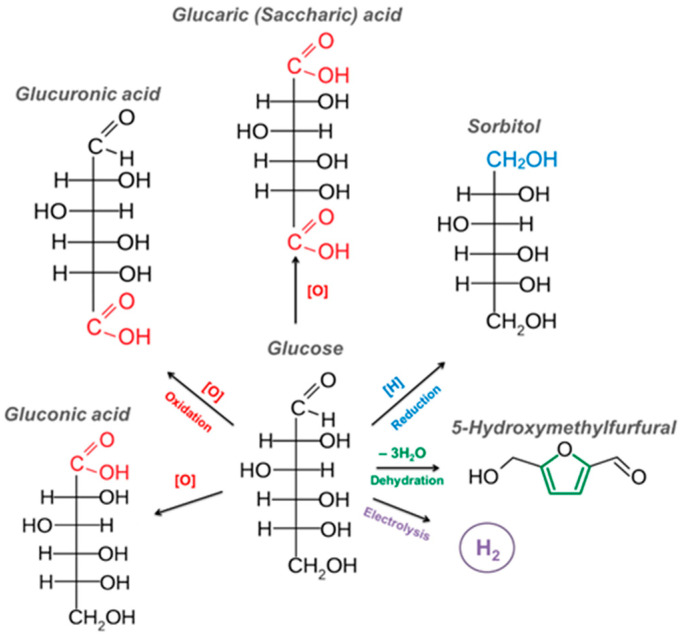
Industrially significant products obtained from glucose.

The most significant ways to convert glucose into value-added products are presented in Figure 3.

The valuable products, obtained from glucose, include gluconic, glucuronic, and glucaric acids, sorbitol, 5-hydroxymethylfurfural, and hydrogen [27,28,29,30,31,32]. In this work, we will dwell at length on the significance and methods of obtaining commercially valuable products of glucose oxidation, gluconic and glucaric acids, based on the analysis of the relevant information on this topic over the last years.

## 2. Gluconic Acid

### 2.1. Product Significance

Gluconic acid (2,3,4,5,6-pentahydroxyhexane acid) is a non-toxic, non-corrosive, mild organic acid, possessing functional properties, with a molecular formula of C_6_H_12_O_7_ [33]. Gluconic acid and its derivatives are found in nature, mainly in plants, fruit juice, grapes, apples, meat, juice, wine, and honey [34].

Gluconic acid and its derivatives have a wide scope of applications and are used in food products as a preserving agent, a marinade, a leavening agent, and a pH control agent (E574-579 food additives). Due to the high ability to form complexes, gluconates are used as a component of juice and fruit puree to prevent turbidity and the formation of calcium and iron phosphates. D-gluconic acid improves the taste of food products, giving them a fresher and less bitter taste. In the dairy industry, gluconic acid prevents the deposition of milkstone in technological equipment and glass storage containers.

D-gluconic acid in the form of calcium (and less often potassium, zinc, and other) salts is widely used in medical practice. Gluconic acid compounds are also used in the production of concrete, cosmetic preparations, agricultural industries, and a number of other industries [35]. D-gluconic acid and its derivatives are used as prebiotics and tend to exhibit antioxidant properties. The chelating properties of gluconates make them a soft and environmentally friendly cleaning agent and degreaser. Glucono-δ-lactone is actively used in cosmetic products [36].

The volume of the gluconic acid market was estimated at 0.2 billion US dollars in 2024. The gluconic acid industry is forecasted to reach 0.28 billion US dollars by 2034, with a combined annual growth rate of 3.5% during the forecast period (2025–2034) [37].

According to Kirimura and colleagues [38], the shares of the use of gluconic acid and its derivatives worldwide by application area are, approximately, construction—45%, food—35%, medical—10%, and other—10% of the industry. Among gluconic acid derivatives in 2023, the largest market segment was occupied by glucono-delta-lactone (40.1%), followed in descending order by sodium gluconate, calcium gluconate, and potassium gluconate [39]. According to the author of the 2025 market research [37], among all segments of the global market, gluconic acid occupies a dominant position in the market, and the fastest growing market segment is glucono-delta-lactone, which is used as an acidifier, leavening agent, and sequestrant in the food industry. The demand for gluconic acid is expected to grow further owing to the increased demand for cosmetics and products containing natural and safe ingredients, including bioacids [39]. In addition, there is growing use of gluconic acid in the agricultural sector as a chelating agent to improve the nutrient uptake by plants and in water treatment to remove heavy metals. The fields of application of gluconic acid and its derivatives are presented in more detail in Table 1.

### 2.2. Gluconic Acid Production

The industrial method of obtaining gluconic acid is currently the microbiological oxidation of glucose using cell cultures [44]. However, it is also worth highlighting other methods of obtaining gluconic acid from glucose:

- Catalytic, using noble metals;
- Electrocatalytic, using electrodes based on noble metals;
- Photocatalytic;
- Enzymatic, using immobilized enzymes.

#### 2.2.1. Microbiological Synthesis

**Figure 4:**
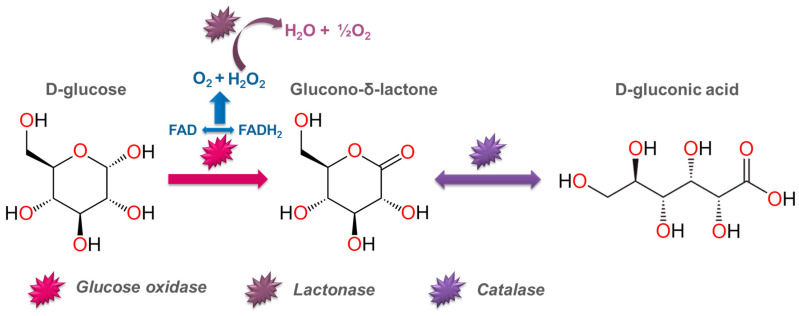
Enzymatic conversion of glucose by means of *Aspergillus niger*.

The biotechnological method is a widely used industrial method of producing gluconic acid using living cell cultures. Various microorganisms and molds capable of producing the enzyme glucose oxidase have been studied in this process: *Aspergillus niger*, *Aureobasidium pullulans*, *Penicillum* spp., bacteria of the *Pseudomonas*, *Gluconobacter*, *Zymomonas mobilis* species, etc. Both pure glucose and a complex nutrient medium (breadfruit hydrolysate, sugarcane molasses, grape must, corncob enzymatic hydrolysate, and others) can be selected as a carbon source for microorganisms [86,87,88]. Figure 4 presents a scheme of the enzymatic conversion of glucose in the presence of Aspergillus niger. Table 2 shows the most commonly used microorganisms for producing gluconic acid and the process characteristics [89].

Microbiological synthesis is the main industrial method for obtaining gluconic acid. It is based on the catalytic activity of the enzyme glucose oxidase, expressed by various microorganisms, which oxidizes D-glucose with atmospheric oxygen to glucono-δ-lactone. Glucono-δ-lactone is subsequently hydrolyzed into gluconic acid.

- Key producers:
  – *Aspergillus niger*: The most widely used fungus, showing high productivity (yields up to 311 g/L [89]) on various substrates.
  – *Gluconobacter oxydans*: A bacterium known for its fast kinetics and high efficiency, especially at certain pH and with the use of neutralizing agents [60,88].
  – *Penicillium* spp.: Various species are also capable of synthesis, adapting to different substrates and conditions [95,96,97].
  – Others: *Aureobasidium pullulans* [91], *Zymomonas mobilis* (including immobilized forms) [98], *Klebsiella pneumoniae* [94], etc., have also been studied.
- Carbon sources:
  – Pure glucose: Used to achieve high titers (yield) but increases the cost of the process.
  – Complex media: Hydrolysates (breadfruit, corn cobs), molasses, must (grape, banana), puree, etc., reduce the cost but can complicate the purification.
- Fermentation conditions and modes:
  – Parameters: Typically mesophilic temperature (≈30–39 °C), controlled pH (often 5.0–6.5, strain-dependent), aeration (1–3 vvm), and agitation.
  – Modes: Batch, fed-batch (often gives best results in terms of gluconic acid yield >140 g/L for *A. niger* [87]), submerged fermentation.
- Product yields vary widely (from ~15 g/L to >300 g/L). Glucose conversion efficiencies are often high (>85–95% of theoretical).

The microbiological synthesis of gluconic acid is a well-studied and industrially developed process. Aspergillus niger and Gluconobacter oxydans remain key producers due to their high efficiency. The use of a variety of substrates, including low-cost ones, and optimized fermentation regimes (especially fed-batch) allow high titers of the product to be achieved. However, as mentioned earlier [27,99,100,101,102] and confirmed by the data in Table 2, there remain challenges associated with the relatively slow reaction rate compared to chemical methods, such as long fermentation times in many cases, low yield per time unit, the generation of significant wastewater volumes, difficulties in isolating the product from complex culture media, and the utilization of waste biomass. Ongoing research is aimed at selecting or engineering more productive strains, optimizing cultivation conditions, and developing more efficient downstream processing methods to overcome these limitations.

#### 2.2.2. Heterogeneous Catalytic Oxidation of Glucose

Another method that allows the disadvantages of the traditional method of obtaining gluconic acid to be overcome is the liquid-phase oxidation of glucose with molecular oxygen in the presence of nanosized heterogeneous catalysts based on noble metals (platinum or palladium) [27,103,104,105]. However, during catalysis in the presence of air or molecular oxygen, these systems are subject to oxidative poisoning; therefore, a modifying metal (Cu, Bi, Te, etc.) is introduced as an additive [106,107,108,109,110,111]. A promoter prevents palladium oxidation and increases the gluconic acid yield owing to the electron interaction between an active metal and the promoter metal. This metal is capable of improving the catalytic characteristics of the material [112,113,114]. Activated carbon, titanium oxide, aluminum oxide, silica gel, cerium oxide, etc., are used as support [103,104,105,109,115]. I. Delidovich and colleagues achieved significant success in obtaining gluconic acid using Au/Al_2_O_3_. Even with a relatively low catalyst loading (the molar ratio of glucose to an active component was 17,000: 1), the glucose conversion (97%) and selectivity for the desired product (96%) were close to 100%. The catalysts and reaction conditions of the catalytic process are presented in more detail in Table 3.

Glucose oxidation in the presence of heterogeneous catalysts containing noble metals allows for a significant reduction in the amount of industrial waste, eliminates the use of aggressive chemical compounds, increases environmental safety, and simplifies the process of separating catalysts from the liquid reaction medium for repeated use. Despite the undoubted advantages of using heterogeneous catalysts, they have a number of disadvantages. The limiting factor in their use at present is the high cost of these materials. The diffusion of reactants to active centers and the diffusion of products from them can be limited, which slows down the reaction rate. A serious problem limiting their use is catalyst deactivation. Even when using a support, the leaching of active metals into the solution can reduce the overall efficiency of the catalytic system [110,121,122,123,124,125]. Strict control of the pH of the reaction medium is also necessary since the formation of gluconic acid proportionally reduces the acidity of the medium. Thus, the catalytic process of glucose oxidation in the presence of heterogeneous supported catalysts is complicated. With a significant increase in the acidity of the medium to pH 11–12, the base-catalyzed reaction of glucose isomerization occurs, as well as the destruction of the reaction products, which leads to a significant decrease in selectivity for the target product (sodium gluconate) [111,126,127].

Gold supported on metal oxides, carbon materials, or as a bimetallic nanoparticle together with other noble metals with the addition of Bi, Sn, or other promoters is generally more stable, while Pt or Pd on carbon supports or some metal oxides may be less stable. The correct choice of support, synthesis method, use of bimetallic catalysts, and optimization of reaction conditions are important conditions for obtaining highly active and stable catalysts. Despite the fact that there is already a lot of information about the reaction of the heterogeneous selective oxidation of glucose, research on optimizing the characteristics of the catalyst to achieve high efficiency and stability in this process is still relevant.

#### 2.2.3. Glucose Electrooxidation

Glucose electrooxidation is a process that is primarily aimed at generating electricity in a low-temperature glucose fuel cell [128]. However, the formation of gluconic acid in the electrode cell allows the electrolytic oxidation of glucose to be considered as a potentially possible method for producing gluconic acid [129]. Gluconic acid is most often not the only product of glucose electrolysis; it is formed together with glucaric acid. Electrodes, based on noble metals (Au, Pt, Pd), as well as electrodes that do not contain noble metals (for instance, MnO_2_/Ti), were studied as electrocatalysts for the process of producing gluconic acid (Table 4) [100,129,130,131].

Despite the high conversion of glucose in a short reaction time and the relative environmental friendliness and simplicity of the process, this method is characterized by low selectivity for the target product (Table 4) [100].

#### 2.2.4. Photocatalytic Oxidation of Glucose

Heterogeneous photocatalysis is a new and promising strategy for the green synthesis of gluconic acid, which attracts much attention because this process can efficiently proceed under the action of UV and visible light in mild conditions at temperatures not exceeding 303 K [121,132,133,134,135]. Photocatalysts are used as substitutes for glucose oxidase, which induces glucose oxidation [132].

In recent years, metallothioporphyrazines (MPzs) have been considered biomimetic photocatalysts due to their strong absorption in the visible region [136]. These systems exhibit unique photocatalytic activity for the activation of hydrogen peroxide or oxygen in visible light owing to the presence of delocalized π-electrons [137]. Such compounds have proven themselves in selective organic transformations in mild conditions [138]. The studies of Cheng M. noted that the application of cobalt thioporphyrazine (0.5%) on ZnO increased the conversion of glucose compared to pure ZnO, 4.4 times in 5 h of the reaction. In relation to gluconic acid, the selectivity increased up to 15% as compared to 7%, achieved when using pure ZnO. At the same time, the total selectivity was below 100%, probably due to the glucose mineralization up to CO_2_ and H_2_O [128]. Yin J. and colleagues obtained a photocomposite material by modifying TiO_2_ with HPW (phosphotungstic acid) and CoPz (cobalt tetra(2-hydroxymethyl-1,4-dithiin)porphyrazine), which demonstrated a selectivity for gluconic acid of 65.5% with a total glucose conversion of 22.2%. In this case, the total selectivity was 80.4%, which also confirms the complete oxidation of glucose to carbon dioxide and water [139]. In the work of Q. Znang, the SnO_2_/FePz(SBu)_8_ composite was obtained by immobilizing tetra(2,3-bis(butylthio)maleonitrile)porphyrazine of iron (FePz(SBu)_8_) on the SnO_2_ surface. The study of this material in the photocatalytic process of glucose oxidation to gluconic acid showed a conversion of 34.2% with a selectivity for the target product equal to 32.9% [140]. In another work, Q. Zhang et al. synthesized the g-C_3_N_4_/CoPz composites by applying cobalt tetra(2,3-bis(butylthio)maleonitrile)porphyrazine onto the surface of graphite-like carbon nitride. The selectivity of glucose in the presence of g-C_3_N_4_/CoPz was 65% with a glucose conversion of 65% and a light intensity of 2 W/cm^2^ [141]. R. Chen et al. applied tetra(2,3-bis(butylthio)maleonitrile)porphyrazine (FePz(SBu)_8_) on a Na-ZSM support in the amount of 5%. The glucose conversion reached 21.7% with a selectivity for gluconic acid of 34.1%. However, the total selectivity for all the products was only 65.7% [136].

**Figure 5:**
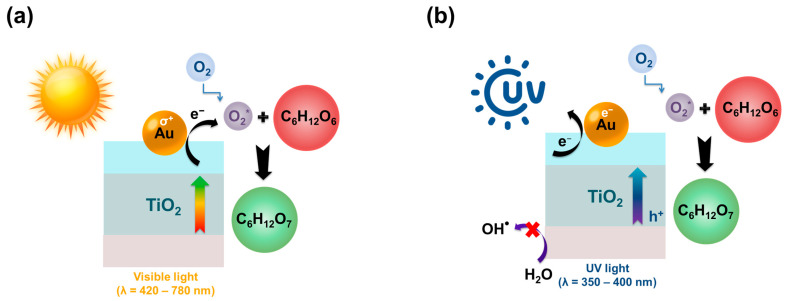
The scheme of highly selective glucose oxidation into gluconic acid under the action of (**a**) visible light and (**b**) UV radiation.

X. Bai reported the efficiency of a metal-free photocatalyst consisting of nitrogen-deficient carbon nitride (BNCN) and chlorin e6 (Ce6) during the oxidation of glucose to gluconic acid. The resulting photocatalyst showed a glucose conversion of 62.3% with a selectivity for gluconic acid of 59%. The total selectivity for arabinose and gluconic and glucaric acids did not exceed 71% [142]. TiO_2_-based photocatalysts are used in photocatalytic processes of oxidation of organic substances, including glucose, owing to the peculiarities of the TiO_2_ band structure [143]. J. C. Colmenares and co-authors succeeded in increasing the total selectivity of the obtained TiO_2_ in relation to gluconic and glucaric acids and arabinose (S_total_ = 71.3%) as compared to the commercial sample of Degussa P-25 (S_total_ = 17.2%) [135]. The authors attributed the improved performance of the photocatalyst to the physicochemical properties (for example, to the high specific surface area, nanostructured anatase phase, and visible light absorption) of the new TiO_2_ materials and the reaction conditions [135]. K. Roongraung and colleagues studied the influence of applying 20% TiO_2_ on Y-type zeolites with different ratios of SiO_2_:Al_2_O_3_. The strength and number of acid sites have been shown to influence the catalytic properties of various catalysts [134]. An increase in the strength of acid sites with increasing aluminum content at SiO_2_:Al_2_O_3_ = 10 led to an increase in the yield and selectivity (29%) of gluconic acid, whereas at SiO_2_:Al_2_O_3_ ratios of 100 and 500, the selectivity was at the level of 11% and 10%, respectively. In addition, L Da Vià and colleagues studied different concentrations of Ag deposited on the photoactive TiO_2_ material during glucose oxidation. The selectivity for gluconic acid was 15–18% with the highest glucose conversion of 11.5% [144]. B. Zhou and coauthors found that Au/TiO_2_ contributed to an increase in the gluconic acid yield in both the visible light region (99%) and the UV radiation region (99%) in an aqueous Na_2_CO_3_ solution with a glucose conversion of 99% in both cases [145]. The authors attributed the increased photoactivity of the catalyst in the visible spectrum to the plasmon resonance of Au nanoparticles. In UV light, Au nanoparticles enhance the photoexcitation of the TiO_2_ band gap, promoting an increase in activity and, as a result, an increase in the gluconic acid yield. Na_2_CO_3_ acts as an inhibitor of reactive oxygen species with a strong oxidizing ability under UV light (for instance, hydroxyl radicals and singlet oxygen). Figure 5 shows the proposed mechanism of glucose photooxidation in the UV and visible regions of the spectrum [145].

Therefore, despite the various approaches to the photocatalytic production of gluconic acid from glucose used by the researchers, the main limitations of this method are still low selectivity for the desired product, losses associated with partial oxidation of glucose to CO_2_ and H_2_O, and the use of expensive gold catalysts [146].

#### 2.2.5. Glucose Oxidation in the Presence of Immobilized Glucose Oxidase

A relatively new method of obtaining gluconic acid is using glucose oxidase immobilized on supports, including magnetically separated ones. In this review, emphasis was placed on the use of immobilized enzymes, since the use of enzymes in their native form is often hampered by a number of limitations, such as high cost, low functional stability, inactivation by physical and chemical factors, and a lack of recovery or reuse [147,148,149,150,151,152]. Therefore, in recent years, many researchers have considered immobilization as an effective tool to overcome these drawbacks and improve the catalytic properties of enzymes, such as activity, selectivity, specificity, and resistance to inhibitors [153]. A biocatalytic method for the oxidation of D-glucose to D-gluconic acid using glucose oxidase immobilized on inorganic supports and magnetically separated systems is a promising direction in the field of biotechnology. Over the past decade, there has been a significant increase in the attention of researchers to magnetic nanoparticles (MNPs) and materials based on them. Firstly, due to the nano-size of the particles, a large surface area of the biocatalyst is achieved, and this significantly increases the probability of contact between the support and the enzyme. In turn, this feature allows the biocatalyst to achieve indicators close to the native enzyme. Secondly, the obtained magnetically separable systems can be easily separated by a magnet and reused for successive cycles [154].

**Figure 6:**
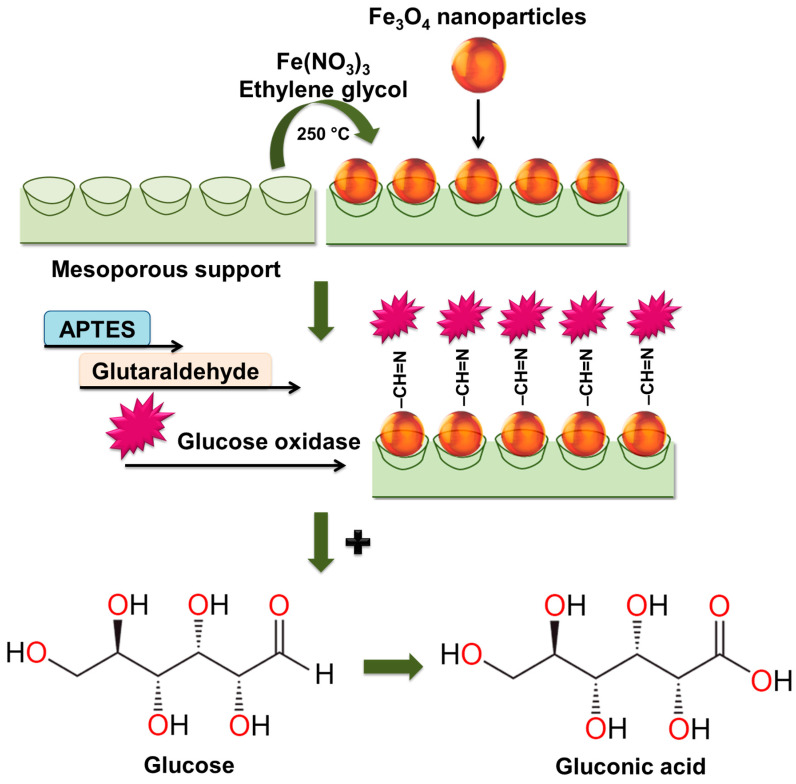
The scheme of the synthesis of the biocatalyst based on glucose oxidase immobilized onto the magnetically separable support and the oxidation of glucose to gluconic acid.

The synthesis of the biocatalyst involves treating a mesoporous support (SiO_2_, Al_2_O_3_, ZrO_2_) with iron nitrate (III) and calcination to form Fe_3_O_4_ nanoparticles [155,156]. The mixed support is functionalized with 3-aminopropyltriethoxysilane (APTES) to modify the support with amino groups, then glutaraldehyde is used for the covalent crosslinking of glucose oxidase with the support surface. Jaquish R. and co-authors found that the treatment of mesoporous SiO_2_ and Al_2_O_3_ with the iron nitrate (III) solution not only imparts magnetic properties but also increases relative activity during gluconic acid production, using the biocatalyst in all 10 consecutive cycles [155]. The improvement in catalytic activity is associated with the enzyme-like properties of iron oxide. However, SiO_2_-based samples showed higher activity compared to that of Al_2_O_3_ used as a support. The authors explain this phenomenon by the larger pores in Fe_3_O_4_-SiO_2_ as compared to those of Fe_3_O_4_-Al_2_O_3_ and stronger Brønsted acid sites. A. K. Haskell and colleagues showed that the highest relative activity of the conversion of glucose into gluconic acid in the presence of the obtained Fe_3_O_4_/ZrO_2_/GOx biocatalyst was 98% when the pH was 6–7 and the temperature was 313–318 K [156]. The research of V. Matveeva et al. described a method of obtaining a biocatalyst that is based on glucose oxidase immobilized on a magnetically separable Fe_3_O_4_/SiO_2_ support [154]. The Fe_3_O_4_/SiO_2_/GOx biocatalyst demonstrated the highest yield of gluconic acid (88%) at an initial glucose concentration of 3.68 mmol/L (the pH was 5, T was 313 K) for 1 h of the reaction process. O. V. Grebennikova and colleagues found that Fe_3_O_4_/SiO_2_/GOx not only has a relative activity of 95% but also exhibits stability in 10 consecutive cycles of glucose oxidation [157]. The work [157] also established that the use of aluminum oxide for the preparation of a magnetically separable support led to a decrease in the relative activity, which in the 10th cycle of the reaction process was equal to ~80%. The combined scheme of the process of preparing the magnetically separable support, the immobilization of glucose oxidase, and the oxidation of glucose to gluconic acid is presented in Figure 6 [155,156,157].

In all the considered cases of using immobilized enzymes applied onto magnetically separable supports, the process was characterized by 100% selectivity. The main advantages of using immobilized enzymes applied onto magnetically separable supports are high enentio-, regio- and chemoselectivity, a high rate of the conversion of glucose into gluconic acid, and the possibility for the rapid separation of the biocatalyst from the reaction medium for its subsequent multiple uses [155,156,157].

Covalent binding is one of the most common and interesting methods of enzyme immobilization for industrial applications. The main disadvantages of the method include the labor-intensive nature of the biocatalyst production and the likelihood of enzyme inactivation in some cases [153]. It should be noted that the cost of most industrial enzymes is often only a minor component in the overall economics of the process. Therefore, in these cases, the additional costs associated with the immobilization of enzymes and the process are often not justified, which limits the widespread use of immobilized enzymes for now [158].

## 3. Glucaric Acid

### 3.1. Product Significance

D-glucaric acid (2,3,4,5-tetrahydroxyadipic acid) is a dibasic acid obtained directly by the oxidation of D-glucose. Glucaric acid is not a widely available chemical product, and the available information on its production and market trends is limited. In recent years, there has been growing interest in glucaric acid as a renewable and eco-friendly chemical feedstock, which may contribute to its increased production in the future. From 2004, Glucaric acid was classified as a “top value-added chemical from biomass” by the United States Department of Energy because of its potential applications as a material for making biodegradable detergents and biodegradable polymers such as nylons and plastics [159], and its market value will be 1.4 billion US dollars by 2028 according to some estimations [27]. However, the selective production of glucaric acid is difficult; therefore, the market for glucaric acid is underdeveloped due to limited availability and high prices [160].

Glucaric acid has chelating properties owing to the presence of two carboxyl groups. This peculiarity of glucaric acid allows it to extract heavy metals, such as Cd, Cr, Cu, Ni, Pb, and Zn, from soil contaminated with wastewater [161]. Glucaric acid is used to create biodegradable polymers for the “green” industrial production of compounds, based on heavy metals and their derivatives after the extraction from contaminated aquatic environments [162]. The chelating ability of glucaric acid allows it to be used for the degradation of organic pollutants [163]. Glucaric acid also serves as a monomer for synthesizing organic biopolymers, such as polyglucaramides, hyperbranched polyethers, and chemically soluble cross-linked gels [164,165,166,167,168]. D-glucaric acid is a non-toxic compound that is produced in small quantities by mammals and some plants. D-glucaric acid is a natural metabolite of D-glucuronic acid conversion in mammal organisms [169]. This feature allowed glucaric acid to be used as a biomarker for early cancer diagnostics and the noninvasive imaging of tumor necrosis [170,171,172,173,174]. Glucaric acid is used as a dietary supplement to regulate the human endocrine profile, improve the immune response, treat diabetes, lower cholesterol, and reduce canceration risk [159,175,176]. Glucaric acid is known to be used as an anticancer chemotherapy [173]. The anticarcinogenic properties of glucaric acid and its lactones are related to the fact that D-glucarate inhibits the action of β-glucuronidase and promotes the elimination of potentially toxic compounds that increase the risk of developing various types of cancer [177,178].

Sodium and calcium glucarates are used as detergents and cleaning agents, while magnesium and calcium salts of glucaric acid are used in hard water treatment and in preventing soap formation [27]. Glucaric acid is a bio-based building block for the synthesis of adipinic acid by the catalytic reduction with hydrogen as well as 2,5-furandicarbonic acid [27,179,180]. Adipinic acid is a precursor to nylons and is used in coatings and detergents [27], while 2,5-furandicarbonic acid is a promising bioplastic monomer that is a potential replacement for terephthalic acid in polyethylene terephthalate [180].

**Figure 7:**
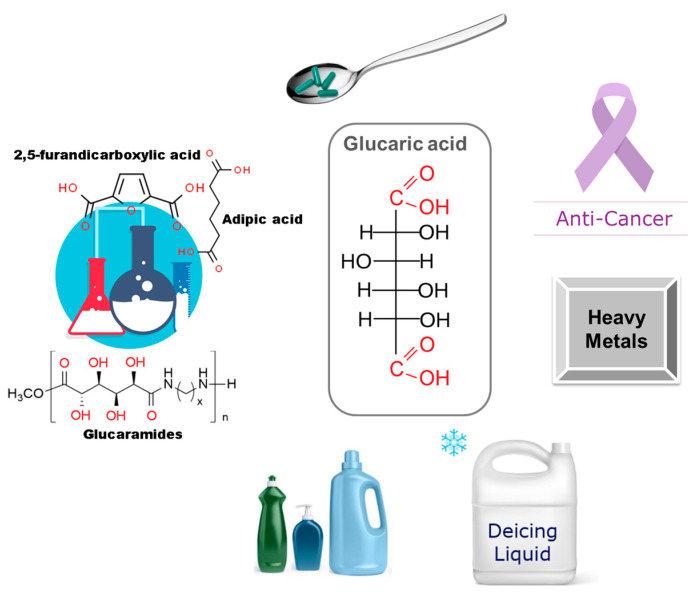
Main applications of glucaric acid: a chemical platform for the synthesis of new compounds, a pharmaceutical food additive, cancer diagnosis and treatment, a heavy metal accumulator, anti-corrosion deicing fluids, and a cleaning agent.

Glucaric acid has anticorrosive, antiplasticizing properties and can be used as a deicing agent. Liquid deicing chemicals usually contain aggressive chloride-based components that can cause significant damage to both road surfaces and the vehicles in which they are used. Phosphates were traditionally used as corrosion inhibitors in deicing mixtures, but their use was hampered by growing environmental concerns [177]. Replacing environmentally harmful phosphates and chlorides with gluconic acid has contributed to the growth of the commercial production of deicing mixtures, based on glucaric acid. The anticorrosive effect is achieved even at sufficiently low concentrations of glucaric acid [27,177,181]. The main areas of using glucaric acid are presented in Figure 7.

### 3.2. Glucaric Acid Production

The synthesis of glucaric acid, as well as gluconic acid, from glucose or glucose-containing raw materials can be accomplished using chemical, catalytic, electrochemical, or biochemical methods. However, only two methods have been practically applied at present [27,182,183]: the chemical oxidation of glucose using nitric acid as an oxidizing agent and oxidation using palladium or platinum catalysts.

#### 3.2.1. Biotechnological Methods of Obtaining Glucaric Acid

The process of the biotransformation of glucose into glucaric acid (glucaric acid = GRA) proceeds with low selectivity. This method of obtaining glucaric acid is characterized by difficulties associated with the separation of products (large volumes of microbial biomass and hundreds of by-products with similar properties are formed) and is not used for industrial production [32].

There are two main routes for glucaric acid biosynthesis. The first involves the introduction of heterologous glucaric acid synthesis pathways into *Escherichia coli* or yeast cells, and the second involves the use of multienzyme biocatalytic methods in vitro [184].

Innovative approaches to the biotechnological production of GRA using *Escherichia coli* and *Pseudomonas syringae* are developing, demonstrating the growing capabilities of modern metabolic engineering and synthetic biology [185,186,187,188]. Thus, a synthetic method was developed to produce glucaric acid from glucose in recombinant *E. coli* using enzymes from three different sources: Ino1, MIOX, and Udh [185]. Ino1, isolated from *Saccharomyces cerevisiae*, converts glucose-6-phosphate to myo-inositol-1-phosphate, which is then dephosphorylated to myo-inositol. MIOX from mice catalyzes the formation of D-glucuronic acid from myo-inositol, which is then converted to D-glucaric acid by Udh from *Pseudomonas syringae*. The activity of this recombinant enzyme was more than two orders of magnitude higher than that of Ino1 and MIOX, allowing the glucaric acid to be obtained at concentrations exceeding 1 g/L. The main limiting factor is the activity of MIOX and the need to optimize enzyme expression [185]. To improve the efficiency of the GRA production by this method, the authors [186] proposed using polypeptide scaffolds from domains of the protein–proteinous interaction to colocalize all three enzymes in a designed complex to synthetically increase the effective concentration of myo-inositol. Since the catalytic activity of myo-inositol oxygenase (MIOX) is highly dependent on the concentration of the myo-inositol substrate, the scaffolds directly increased the specific activity of MIOX and the glucaric acid titers correlated with MIOX activity. An approximately fivefold increase in GRA titers was observed as compared to the scaffold-free control and a 50% increase as compared to the previously reported highest titers. To increase the productivity of this pathway, protein fusion tags that increase the solubility of MIOX and directed evolution to increase the activity of MIOX were studied [187]. The fusion of the N-terminal fragment of SUMO to MIOX increased the final titers of D-glucaric acid to 4.85 g/L from 10.8 g/L of myo-inositol and the increase in the production of glucaric acid from myo-inositol was 75%. The expression of a small fragment of manXmRNA allowed the final titer of D-glucaric acid to reach 4.58 g/L with an increase in glucaric acid production of 65%. Using systematic metabolic engineering, the *E. coli* GA10 strain [188] was developed, which allowed a titer of 5.35 g/L to be achieved. Since *E. coli* is not considered a completely safe strain, and with the aim of increasing the yield of GRA, the researchers demonstrated the use of modified *Pichia pastoris* as a platform for the production of glucaric acid from myo-inositol [189]. Glucaric acid with the highest concentration of 6.61 ± 0.30 g/L was obtained synthetically in *Pichia pastoris* by the coexpression of murine myo-inositol oxygenase (mMIOX) and uronate dehydrogenase (Udh) from *Pseudomonas putida* KT2440 during fermentation in a nutrient medium of a mixed substrate containing glucose and myo-inositol. These studies on the use of scaffolds in *E. coli* and the protein fusion strategy in *P. pastoris* provide the basis for further research on increasing the titer of glucaric acid. Attempts were also made to use *Saccharomyces cerevisiae* to obtain D-glucaric acid [178,190,191]. The paper [191] presents a table with the results for 2009–2023 on the production of D-glucaric acid using *E. coli*, *S. cerevisiae* yeast, or *P. pastoris* in vivo. The highest titer of D-glucaric acid at a level of 11–13 g/L was shown to be achieved using *Saccharomyces cerevisiae*. Tables with the results of using biocatalytic methods can also be found in the works Chen L.-Z. et al. and Zhao Y. et al. [159,184]. The development of biotechnological methods for the production of glucaric acid promises to make it an affordable and environmentally friendly alternative to chemical methods.

#### 3.2.2. Chemical Methods of Obtaining Glucaric Acid

**Figure 8:**
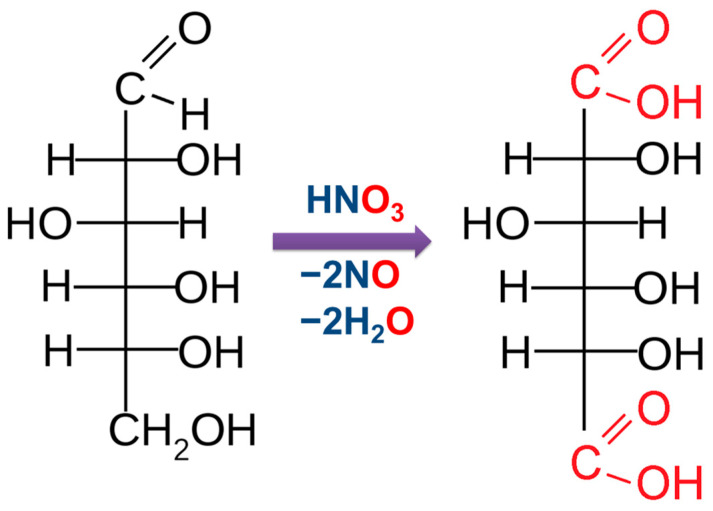
The obtainment of glucaric acid by glucose oxidation.

When exposed to strong oxidizing agents, both the aldehyde group of glucose and the primary alcohol group undergo oxidation. In this case, dibasic glucaric acid is formed (Figure 8).

The oxidation of glucose with nitric acid in the absence of catalysts, followed by the obtainment of glucaric acid at temperatures from plus 328 K to plus 348 K, is described in the work [192,193]. Since D-glucaric acid is not easily crystallized, it is convenient to isolate salts (calcium or potassium glucarates) by neutralizing the bases with glucaric acid. Despite its commercial potential, the large-scale production of D-glucaric acid by the oxidation of D-glucose with nitric acid is difficult, mainly because of competing side reactions, resulting in a low degree of conversion into D-glucaric acid, and the rapid and highly exothermic nature of the oxidation [194]. This method remains attractive for commercialization owing to its relative simplicity, since nitric acid serves as both a solvent and an oxidizing agent in the synthesis, and because of the low cost of the oxidizing agent, although the product yield is only 40–45%. In the process, implemented by Rivertop Renewable, nitric acid is oxidized at temperatures from 298 to 313 K and a total pressure from 1.25 to 1.5 bar, with a yield of 35 to 40% [27]. Significant disadvantages of this method are significant costs in organizing the production and the formation of toxic by-products and inorganic salts, which worsen the ecology [194].

#### 3.2.3. D-Glucose Oxidation to Glucaric Acid Using Catalysts

There are known works [195,196] reporting on the homogeneous catalyzed oxidation of glucose by means of HNO_3_ and NaNO_2_. Studying the glucose oxidation process in the temperature range from plus 298 K to plus 313 K, the authors of [194] showed that nitric acid is, in fact, a catalyst in this process, and oxygen is consumed for oxidation. Sodium nitrite catalyzes the sequential oxidation of D-glucose with oxygen in the strongly acidic solution of HClO_4_-H_2_O-sulfolane to D-gluconic and then to D-glucaric acids [195].

Other catalytic systems are also being developed. D-glucose can be oxidized with sodium hypochlorite (potassium hypochlorite) in the presence of sodium (potassium) bromide using 2,2,6,6-tetramethylpiperidine-1-oxyl (TEMPO) or 4-acetamido-2,6,6-tetramethylpiperidine-1-oxyl (4-acetamido-TEMPO) as a catalyst [196,197]. When the pH values range from 11.4 to 11.6 and the temperature is below 5 °C, the D-glucose oxidation, initiated by nitroxide, produces glucaric acid salts with high selectivity and a good yield of more than 85%.

In 2001, a work appeared on the catalyzed 4-acetylamino-2,2,6,6-tetramethyl-1-piperidinyloxy (4-AcNH-TEMPO) oxidation of D-glucose to D-glucaric acid using elemental chlorine or bromine as a final oxidizer at a temperature of 273–278 K and a pH of 11.5 [198]. Glucarate yields of over 90% were reported. Unfortunately, 4-AcNH-TEMPO is expensive to produce and there are problems with its utilization, which hinders the widespread use of this catalyst [199]. The disadvantages of such processes, apart from using corrosive and hazardous reagents, also include difficulties in separating and utilizing homogeneous catalysts from the products. Gluconic, glucaric, and 2-keto-gluconic acids and their salts can be obtained by the oxidation of aqueous glucose solutions with oxygen, air, or hydrogen peroxide [200] in the presence of metal catalysts on supports. During the synthesis of gluconic acid, the catalysts containing gold are preferable as catalysts, and catalysts containing platinum on various supports (TiO_2_, ZrO_2_, C) are desirable in the case of glucaric acid synthesis [27].

Glucose is oxidized to gluconic acid faster than gluconic acid is oxidized to glucaric acid. For instance, the work [201] noted that after 10 min, a high conversion of glucose (about 64%) with gluconic acid as the main product is achieved when using a Pt/C catalyst with a selectivity of about 81%. After 10 h of glucose oxidation, its conversion is observed with a selectivity for glucaric and gluconic acids of 65% and 19%, respectively. The hydroxyl group (-OH) is oxidized on C6 of gluconic acid, accompanied by the formation of an intermediate product (glucuronic acid), which is then oxidized to form glucaric acid. The yield of glucaric acid is lower than that of gluconic acid because the oxidation of the -OH-group of gluconic acid is thermodynamically less favorable than the oxidation of the aldehyde group of glucose [28]. The selectivity in relation to glucaric acid is influenced by side and consecutive reactions of the C−C bond cleavage.

In view of this, the reaction of the direct conversion of glucose into glucaric acid is still a relevant technological task due to the formation of a number of undesirable products that reduce the glucaric acid yield. Based on the analysis of the literature data, a recent review article [27] provides a table containing the catalyst type used by researchers in the reaction to obtain glucaric acid and the process conditions, taking into account the obtainment of the final product in one reactor. The best results were obtained using the 5% Pt/CNT catalyst (the conversion was 100%, the selectivity was 82%, and the glucaric acid yield was 82%). The catalysts that contained gold and gold–palladium showed the worst results. For instance, for the 3.5% Au−3.45% Pt/ZrO_2_ catalyst, during the conversion of 100%, the selectivity and the yield of glucaric acid were only 50%.

The degree of conversion, the selectivity of the process, and the glucaric acid yield depend on the conditions of the oxidation process, such as temperature, the partial pressure of oxygen (PO_2_), the initial concentration of glucose, the time, and the pH of the reaction medium.

The highest yield of glucaric acid is observed when the reaction proceeds in a neutral or slightly alkaline medium. The assessment of the influence of the partial pressure of oxygen on the yield of glucaric and gluconic acids in the reaction of glucose oxidation on the various catalysts under review [27] showed an increase in the yield of gluconic and glucaric acids when the partial pressure increases from 1 to 13.8 bar. When obtaining glucaric acid in sequential operation reactors, the first stage of the reaction can be conducted at a lower pressure, which increases the process efficiency.

As for the temperature of the process, it varied in the studies of different researchers from 333 to 353 K, and the highest yield of 82% was recorded at 333 K [202].

In comparison to chemical methods, glucose oxidation in the presence of heterogeneous catalysts containing noble metals seems to be preferable. When using this method, the amount of industrial waste is significantly reduced. There is no need to use aggressive chemical compounds, environmental safety is increased, and the process of separating the catalysts from the liquid reaction medium and their repeated use is simplified. Despite the fact that there is already much information on the reaction of the heterogeneous selective oxidation of glucose, the problem of optimizing the catalyst composition to achieve its high efficiency and stability in this process still remains unresolved.

Using the photocatalytic method of glucose oxidation to obtain gluconic and glucaric acids and a number of other products is known [139,140]. This review examines the latest published works on this topic.

Using SnO_2_/FePz(SBu)_8_ as a photocatalyst, the photocatalytic oxidation of glucose in water in aerated conditions upon exposure to light of the visible spectrum was reported [140]. Metallothioporphyrazines (MPzs), which include sulfur-containing groups at the periphery of the porphyrinic macrocycle, have an extensive system of delocalized π-electrons and are characterized by strong absorption in the visible region of light, which can improve the photocatalytic activity of the catalysts. When conducting the oxidation process with SnO_2_, the glucose conversion was only 6.4% with a selectivity of 28.7% for gluconic acid, while, when using FePz(SBu)_8_, the conversion was less than 0.1% and trace amounts of the products were observed. It turned out that a synergistic effect was observed when using SnO_2_/FePz(SBu)_8_, and it was possible to achieve a 34.2% conversion of glucose with a selectivity of 32.9% for gluconic acid and 12.9% for glucaric acid. In addition to these acids, formic acid and other organic acids were also formed. To achieve highly selective oxidation of glucose to gluconic and glucaric acids, the authors of [139] prepared a new composite TiO_2_/HPW/CoPz photocatalyst by modifying TiO_2_ by means of HPW (phosphotungstic acid) and CoPz (cobalt tetra (2-hydroxymethyl-1,4-dithiine)porphyrazine). It has been shown that for the photocatalyst of the TiO_2_/HPW(29%)/CoPz(1%) composition, the glucose conversion degree in the oxidation process under the action of light in the visible region of the spectrum is 22.2%, and the selectivity for gluconic acid is 63.5% and 16.9% for glucaric acid.

For the selective oxidation of glucose to glucaric acid, a photoanode was developed, which is a single-atom Pt anchored on defective TiO_2_ nanorod arrays. The high selectivity of the glucose oxidation process is achieved by optimizing the oxygen vacancies of the defective TiO_2_ photoanode. Using such a photoanode allowed an 84.3% yield of glucaric acid to be achieved in 5.5 h during a glucose conversion of 98.8% and a photocurrent density of 1.91 mAcm^−2^ [203].

Summarizing the review of the works devoted to using photocatalysts for the production of gluconic and glucaric acids during glucose oxidation, one can note that using solar energy, inexpensive, non-toxic metal oxide semiconductors, and mild conditions for the glucose oxidation process in an aqueous medium seems attractive. The latest results allow hope for the commercialization of these developments, but the search for an effective photocatalyst for this process is still an urgent task.

#### 3.2.4. The Electrochemical Method of D-Glucose Oxidation to Glucaric Acid

During the electrochemical oxidation of glucose, both glucaric acid and gluconic acid are typically present in the reaction products, and the process is conducted using catalytic systems of various compositions.

To carry out the electrooxidation of glucose to gluconic acid and glucaric acid, Bin et al. [100] used an electrocatalytic reactor with a tubular porous titanium anode coated with nanosized MnO_2_ (MnO_2_/Ti electrode) and a stainless steel mesh as a cathode. The experiments were performed at a glucose concentration in water of 50.5 mol/L, a temperature of 303 K, a current density of 4 mA cm^−2^, and a pH of 7, using 4.98 mass. The percentage of MnO loading showed that, during the oxidation time (19 min), it was possible to achieve 98% glucose conversion and 98% total selectivity for gluconic and glucaric acids (43% selectivity for gluconic acid and 55% selectivity for glucaric acid), which was obtained during electrocatalysis. Increasing the current density up to 6 mA cm^–2^ allowed the conversion degree to be increased up to 99%, the total selectivity up to 99%, and the selectivity for glucaric acid up to 84%. At temperatures above plus 303 K, the selectivity and total conversion of glucose significantly decreased. The authors explain the achieved good results of controlled glucose oxidation by the mass transfer, enhanced by convection, as well as by the timely removal of the desired products from the reactor.

The use of TEMPO in the indirect electrochemical oxidation of D-glucose to glucaric acid in a cell with a graphite felt anode in combination with a stainless steel cathode was reported [204]. It was noted that, by optimizing such parameters as pH (12.2), temperature (plus 278 K), anode type, and the amount of the catalyst, glucaric acid can be obtained with a yield of 85%.

**Figure 9:**
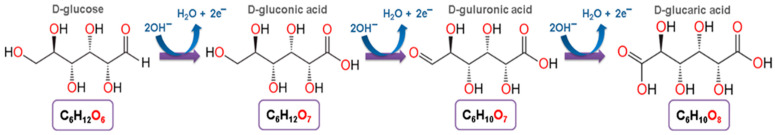
The scheme of the electrochemical oxidation of glucose to gluconic, glucuronic, and glucaric acids.

High glucose conversion and glucaric acid yield in 2 h of the process (98.3% and 83.3%, respectively) were obtained using complex catalytic systems based on nickel and iron (NiFeOx-NF). Faraday’s efficiency in this process was 87% [32]. According to the authors, the electrochemical oxidation of glucose proceeded in several stages: the oxidation of glucose to gluconic acid, in which two electrons are involved, and the oxidation of gluconic acid to glucaric acid, in which four electrons are involved (Figure 9) through an intermediate product (guluronic acid) [32].

The technical and economic analysis conducted by the authors [32] showed that such electrochemical production of glucaric acid is 54% cheaper than production requiring the application of currently used chemical methods.

The two-stage synthesis of D-glucaric acid through D-gluconic acid by the electrocatalytic oxidation of D-glucose on a gold electrode (in a batch operation cell) allowed D-glucaric acid to be obtained with a selectivity of 89.5%. Such high indicators of similar electrocatalytic systems indicate that such oxidation can be conducted effectively, which will be economically advantageous [202].

The parameters of the glucose electrooxidation on the various most promising catalysts, considered above, are provided in Table 5.

Among the considered widely used methods (biochemical, catalytic) of glucose oxidation, electrochemical methods are, in our opinion, the most promising for obtaining glucaric acid, since they avoid the use of aggressive and toxic reagents and reduce the amount of waste. At present, electrochemical technologies have received a new impetus for development, since they satisfy most of the postulates of “green” chemistry, being a clean and carbon-neutral way to stimulate chemical transformations, and they can potentially use peak surpluses of renewable electric energy.

## 4. Conclusions

The processing of biomass waste into glucose and its subsequent conversion into valuable chemical products is a pressing issue in green chemistry. Both gluconic and glucaric acids have unique properties that put them in demand in a wide range of industries—from the food industry and pharmaceuticals to the production of polymers and cleaning agents. Glucaric acid, recognized as a “top value-added chemical”, has particularly high potential, although its market is still less developed.

The production of gluconic acid by biotechnology (*Aspergillus niger*, *Gluconobacter oxydans*) is a traditional industrial process. However, existing methods have limitations in the rate of the biotechnological process, are characterized by the complexity of product isolation, and lead to the formation of wastewater. There are alternative ways to obtain gluconic acid, such as heterogeneous catalysis, electrocatalysis, and photocatalysis, which can potentially increase the rate and simplify the process of obtaining gluconic acid as well as reduce the environmental burden. The main problems are the high cost of catalysts based on noble metals, their stability, and control of the selectivity of the target process. The immobilization of enzymes, such as glucose oxidase, will ensure high selectivity and the possibility of repeated use, especially when immobilized on the surface of magnetic supports. The key limitations are the cost of immobilization and the preservation of enzyme activity under industrial conditions.

The selective production of glucaric acid is much more difficult than gluconic acid. The traditional method of oxidation with nitric acid is ineffective and environmentally unsafe. A promising method for producing glucaric acid is the use of heterogeneous electro- and photocatalysis. However, selectivity remains the main problem due to competing reactions and the breaking of C-C bonds. Nevertheless, the development of catalysts (based on Pt, Au, NiFe, and MnO_2_ photocatalysts) will allow the achievement of high efficiency and potential economic benefits.

Key issues and promising research areas:

1. Increasing selectivity, especially in the synthesis of glucaric acid and using catalytic/photocatalytic methods, is a priority.

2. It is necessary to develop cheaper, more stable, and active catalytic systems (including base metals) and biocatalysts with enzymes (with improved stability and immobilization efficiency).

3. Promising laboratory developments (electro- and photocatalysis, enzymatic catalysis) require scaling and the assessment of technical and economic feasibility. The integration of different approaches is possible (for example, combining electro- or photocatalytic processes with enzymatic ones).

4. It is necessary to adapt the processes to work with real, less pure biomass hydrolysates and develop effective, cost-effective methods for isolating and purifying target products from complex mixtures.

5. Further research in the field of green chemistry, waste minimization, the use of renewable energy sources (especially for electro- and photocatalysis), and the evaluation of the process cycle are integral parts of future developments.

Thus, the conversion of glucose from biomass to gluconic and glucaric acids represents a dynamically developing field with great potential for the sustainable production of valuable chemicals. Overcoming the existing challenges requires an interdisciplinary approach that combines the efforts of chemists, biologists, and engineers, which will allow the full potential of these acids to be realized for the needs of modern industry.

## Figures and Tables

**Table 1:** Applications of gluconic acid and its derivatives in various fields of industry.

Application	Additive	Functional Properties	Ref.
Food industry (food additives: E574–580)	D-gluconic acid (E-574)	Acidity regulator. Raising agent. Gluconic acid reduces the turbidity of dry diluted skim milk.	[34,40,41,42,43,44,45]
	Glucono-δ-lactone (E-575)	Glucono-δ-lactone is a leavening agent for preparing bakery products, as well as for reducing the absorption of fatty compounds. Glucono-δ-lactone is also added to yogurt, cottage cheese, meat, and pickles as an acidity regulator and to tofu for protein coagulation. Sequestering agent.	[34,46,47]
	Sodium gluconate (E-576)	Sequestering agent. Stabilizer. Thickener.	[34]
	Potassium gluconate (E-577)	Acidity regulator. Yeast nutrient. Nutritional supplement.	[34]
	Calcium gluconate (E-578)	Acidity regulator. Hardener. Sequestering agent. Nutritional supplement. Calcium therapy. Animal nutrition.	[34,47]
	Iron gluconate (E-579)	Color preservative. Stabilizer. Nutritional supplement.	[34]
	Magnesium gluconate (E-580)	Magnesium gluconate is used as an acidity regulator in food products. Firming agent. Yeast nutrient. Nutrient supplement.	[34]
Cleaning agents (household chemicals)	D-gluconic acid	Gluconic acid replaces toxic phosphates that are harmful to the environment in dishwashing detergents and washing powders. Gluconic acid is used in water conditioning systems to remove alkaline and biofilms. Gluconic acid effectively cleans mineral deposits, rust, and scale from the surfaces of industrial equipment made of aluminum, steel, and metal alloys.	[34,41,42,43,44,45]
	Sodium gluconate	It is a component of washing powders and household detergents and bleaches, enhances the action of other components, reduces their corrosive properties, and improves solubility in water. It is used in professional alkaline cleaning agents for the industrial removal of organic and inorganic sediment, rust, carbon, and silicate deposits from glass surfaces and the cleaning of aluminum surfaces (e.g., the facades of buildings, aircraft, and containers). Alkaline solutions of sodium gluconate at a temperature of 368–373 K are an effective means for the rapid removal of paint and varnish without damaging the underlying surfaces.	[41,42,43,44,45]
Pharmaceutical industry and medicine	Calcium gluconate	Calcium gluconate in the form of a gel is used to heal burns caused by HCL. Calcium gluconate vaccinations are used for severe cases to avoid deep tissue necrosis, thereby driving industry growth. Calcium gluconate is used as a biologically neutral Ca^2+^ carrier and to replenish calcium deficiency in the human body.	[39,42,45,48]
	Copper gluconate	Copper gluconate is used as a biologically neutral carrier of Cu^2+^ and to replenish copper deficiency in the human body.	[48]
	Potassium gluconate	Potassium gluconate is used as a biologically neutral K^+^ carrier and to replenish potassium deficiency in the human body. Complexes of Na and K gluconates are included in solutions for preserving transplanted organs during their transportation.	[49,50]
	Zinc gluconate	Zinc gluconate is used as a biologically neutral carrier of Zn^2+^ and to replenish zinc deficiency in the human body. Zinc gluconate contributes to strengthening the immune system, relieving cold symptoms and reducing their duration. Zinc gluconate has been shown to be effective in treating skin conditions, including acne, and healing wounds and cuts. Zinc gluconate is used as an ingredient in the treatment of various conditions caused by zinc deficiency, such as delayed puberty, mental sluggishness, skin changes, and susceptibility to infections.	[45,47,48,51,52,53,54]
	Sodium gluconate	Complexes of sodium and potassium gluconates are included in solutions for preserving transplanted organs during their transportation. Sodium gluconate complexes have demonstrated excellent anticancer activity. The in vivo application of gluconate has been found to specifically and irreversibly inhibit pmCiC (plasma membrane citrate transporter), thereby reducing subcutaneous pancreatic tumor growth and changing tissue metabolic characteristics.	[49,55]
	Iron gluconate	Iron gluconate is used as a biologically neutral carrier of Fe^2+^ and to replenish iron deficiency in the human body.	[47,48]
Cosmetics	Gluconic acid	Gluconic acid is used in cosmetics as a divalent metal chelator, a preservative, and a pH regulator and is used in skincare products as a skin-protecting and fragrance agent.	[36,47,56,57,58]
	Calcium gluconate	These gluconates are used in skincare products as a protective and fragrance agent.	[47]
	Potassium gluconate		
	Sodium gluconate		
	Glucono-δ-lactone	Glucono-δ-lactone is used in cosmetics as a divalent metal chelator, a preservative, and a pH regulator. Owing to its large molecule size, glucono-δ-lactone does not penetrate into the deep layers of the skin, thus minimizing the number of side effects. It is used as an active ingredient to combat aging and acne, and it contributes to building a natural protective skin barrier. Glucono-δ-lactone protects the skin from dehydration and free radicals and has a light peeling effect, which makes it suitable for use in skincare products.	[35,46,58]
Building and construction industry	Sodium gluconate	Sodium gluconate is added to cement to better retain the dispersion, to reduce the setting rate of cement mortars, and, as a result, to release less reaction heat, which reduces the risk of cracking when the cement dries.	[59,60,61]
	Gluconic acid	Gluconic acid is used as an additive in cement to control the setting and increase the strength and water resistance.	[44]
Agricultural industry	Calcium gluconate	Calcium gluconate is used as a feed additive for animals, increasing the milk yield in cows and improving the morphology and function of the gastrointestinal tract due to the stimulation of epithelial cell proliferation and the improvement in the intestinal barrier function of livestock. Calcium gluconate promotes weight gain.	[45,62,63,64,65,66,67]
	Iron gluconate	Iron gluconate is a foliar fertilizing agent in gardening.	[45]
	Potassium gluconate	Spraying leaves with potassium gluconate enhances the photosynthesis of leaves in the light and dark phases and promotes the development and intensive growth of seeds of the *Styrax tonkinensis* species. Potassium gluconate enhances oil formation in seeds.	[67]
Biopolymers	Gluconic acid	Gluconic acid acts as a monomer for the synthesis of biodegradable copolymers, such as poly(glycolic acid-co-gluconic acid), poly(L-lactic acid-co-glycolic acid-co-gluconic acid), and poly(acetonide gluconic acid), which can be used for controlled drug delivery.	[68,69,70,71]
“Green solvent”	Gluconic acid	A 50% aqueous solution has been applied as an environmentally friendly catalytic medium for organic synthesis and a “green” solvent.	[72,73,74,75,76]
Textile industry	Sodium gluconate	Sodium gluconate prevents iron precipitation.	[44]
	Glucono-δ-lactone	Glucono-δ-lactone is a fabric-bleaching stabilizer.	[77]
Leather industry	Gluconic acid	A mixture of Fe^2+^ and gluconic acid in a molar ratio of 1:3 promotes the formation of a compound that increases the thermal stability of collagen and the aging time during leather tanning.	[78]
Miscellaneous	Gluconic acid	Low concentrations of gluconic acid are used in water recirculation systems, such as cooling towers and heat exchangers.	[43]
		The addition of gluconic acid to the magnetic paper composition contributes to a reduction in the size of nanomagnetite particles and, as a result, to the manifestation of superparamagnetic properties. Gluconic acid enhances mechanical strength and increases the brightness and transparency of magnetic paper.	[79]
		Gluconic acid is a component of copper-bearing solutions, used in cathodic processes of copper electrodeposition.	[80]
		Gluconic acid exhibits antibacterial properties in fermented teas, including Kombucha.	[81,82,83]
	Sodium gluconate	Sodium gluconate is used as an environmentally friendly component for the galvanic codeposition of nickel-iron alloy. Composites that are based on epoxy resin with precipitated layered double hydroxides, intercalated with gluconic acid anion, have the potential for use as environmentally friendly flame antipyrenes.	[84,85]

**Table 2:** Various microorganisms, process conditions, and gluconic acid yield.

Microorganisms	Carbon Source	Conditions	Yield	Mode	Ref.
*Aspergillus niger*	Breadfruit hydrolysate (120 g/L)	T = 303 K, 72 h, pH of 5.5, agitated at 300 rpm, 2 vvm aeration rate.	109.95 g/L (88.70%)	Batch bioreactor	[86]
*Aspergillus niger* MUM 92.13	Pure glucose (100 g/L)	T = 301 K, 24 h, pH of 6, stirring at 400 rpm, 1 vvm aeration rate, 4 bar air pressure.	70 g/L (0.97 g/g)	Batch stirred tank reactor	[87]
	Sugarcane molasses (ScM): 100 g/L of glucose + sucrose	T = 301 K, 24 h, pH of 6, stirring at 400 rpm, 1 vvm aeration rate, 4 bar air pressure.	78 g/L (1.23 g/g)	Batch stirred tank reactor	
		T = 301 K, pH of 6, stirring at 400 rpm, 1 vvm aeration rate, 4 bar air pressure. One pulse of ScM (to attain 40 g/L of sucrose + glucose in the medium) is added to the batch culture after 48 h of cultivation.	114 g/L (1.3 g/g)	Step-wise fed-batch (1 pulse)	
		T = 301 K, pH of 6, stirring at 400 rpm, 1 vvm aeration rate, 4 bar air pressure. Two pulses of ScM (to attain 40 g/L of sucrose + glucose in the medium) are added to the batch culture after 48 h and 80 h of cultivation.	140 g/L (1.3 g/g)	Step-wise fed-batch (2 pulses)	
	Grape must (GM): 60 g/L of glucose + fructose	T = 301 K, 24 h, pH of 6, stirring at 400 rpm, 1 vvm aeration rate, 4 bar air pressure	36 g/L (1.34 g/g)	Batch stirred tank reactor	
		T = 301 K, pH of 6, stirring at 400 rpm, 1 vvm aeration rate, 4 bar air pressure. One pulse of GM (to attain 15 g/L of glucose in the medium) is added after 24 h to the batch culture	47 g/L (0.91 g/g) 50 g/L (0.80 g/g)	Step-wise fed-batch (1 pulse)	
		T = 301 K, pH of 6, stirring 400 at rpm, 1 vvm aeration rate, 4 bar air pressure. Two pulses of GM (to attain 15 g/L of glucose in the medium) are added after 24 h and 48 h to the batch culture.		Step-wise fed-batch (2 pulses)	
*Aspergillus niger* AN151	Pure glucose (330 g/L)	T = 311 K, pH of 5.5, 14.5 h, 0.1 MPa of pressure. Aeration and agitation rates are set at 1.2 vvm and 550 rpm.	311 g/L (1.05 g/g)	Submerged fermentation	[89]
*Aspergillus terreus*	Pure glucose (122.8 g/L)	T = 301 K, pH of 6.5, 144 h. Aeration and agitation rates are set at 1 vvm and 300 rpm.	92 g/L (0.74 mol/mol·l^−1^)	Batch fermentation	[90]
*Aureobasidium pullulans* NCYC 4012	Pure glucose (80 g/L)	T = 301 K, pH of 6.5, 72 h. Aeration and agitation rates are set at 1 vvm and 400 rpm.	0.48 g/g	Batch stirred tank reactor (conventional)	[91]
		T = 301 K, pH of 6.5, 72 h. Aeration and agitation rates are set at 3 vvm and 600 rpm.	0.76 g/g		
		T = 301 K, pH of 6.5, 72 h. Aeration and agitation rates are set at 1 vvm and 600 rpm. T = 301 K, pH of 6.5, 72 h. Aeration and agitation rates are set at 3 vvm and 600 rpm.	0.78 g/g		
			0.99 g/g		
		T = 301 K, pH of 6.5, 72 h. Aeration and agitation rates are set at 1 vvm and 400 rpm, 1 bar.	0.40 g/g	Batch stirred tank reactor (pressurized)	
		T = 301 K, 6.5 pH, 72 h. Aeration and agitation rates are set at 1 vvm and 400 rpm, 4 bars.	0.42 g/g		
	ScM: 110 ± 10 g/L of glucose + sucrose	T = 301 K, pH of 6.5± 0.5, 96 h. Aeration and agitation rates are set at 1 vvm and 400 rpm at atmospheric pressure.	0.88 g/g	Batch stirred tank reactor (conventional)	[92]
		T = 301 K, pH of 6.5 ± 0.5, 96 h. Aeration and agitation rates are set at 1 vvm and 600 rpm, 1 bar air pressure.	1.08 g/g	Batch stirred tank reactor (conventional)	
		T = 301 K, pH of 6.5 ± 0.5, 96 h. Aeration and agitation rates are set at 1 vvm and 400 rpm, 1 bar air pressure.	0.65 g/g	Batch stirred tank reactor (pressurized)	
		T = 301 K, pH of 6.5 ± 0.5, 96 h. Aeration and agitation rates are set at 1 vvm and 400 rpm, 4 bar air pressure.	0.83 g/g		
		T = 301 K, pH of 6.5 ± 0.5, 168 h. An aeration rate is set at 1 vvm, 1 bar air pressure.	0.61 g/g	Airlift bioreactor	
		T = 301 K, pH of 6.5 ± 0.5, 168 h. The aeration rate is set at 2 vvm, 1 bar air pressure.	0.97 g/g		
*Gluconobacter oxydans* 621H	Corncob enzymatic hydrolysate (100 g/L)	T = 303 K, pH of 2.5, 3 h. Aeration and agitation rates are set at 1.5 vvm and 220 rpm.	72.7 g/L (88%)	Batch stirred tank reactor	[88]
		The same, but the pH is 3.5.	79.6 g/L		
		The same, but the pH is 4.5.	87.3 g/L		
		The same, but the pH is 5.5.	90.3 g/L		
		The same, but the pH is 6.5.	69.0 g/L		
		T = 303 K, pH of 5.5, 36 h. Aeration and agitation rates are set at 1.5 vvm and 220 rpm using CaCO_3_ as a neutralizer	A maximum of 70 g/L at 7 h		
		The same, but using NaOH as a neutralizer.	A maximum of 82 g/L at 7.5 h		
		The same, but using NH_3_·OH as a neutralizer.	A maximum of 63 g/L at 7.5 h		
		The same without any neutralizers.	A maximum of 96 g/L at 24 h		
*Gluconobacter oxydans*	Pure glucose (60 g/L)	T = 303 K, pH of 5.5, 36 h. The agitation rate is set at 220 rpm.	A maximum of 32 g/L at 6 h	Batch fermentation in a shaken flask	[60]
	Pure glucose (120 g/L)		A maximum of 80 g/L at 12 h		
	Pure glucose (180 g/L)		A maximum of 180 g/L at 24 h		
	Pure glucose (240 g/L)		A maximum of 225 g/L at 36 h		
	Pure glucose (300 g/L)		A maximum of 159.5 g/L at 36 h		
	Concentrated enzymatic hydrolysate (CEH): 60 g/L of glucose		A maximum of 7 g/L at 6 h		
	CEH: 120 g/L of glucose		A maximum of 86 g/L at 18 h		
	CEH: 180 g/L of glucose		A maximum of 170 g/L at 36 h		
	CEH: 240 g/L of glucose		A maximum of 140 g/L at 36 h		
	CEH: 300 g/L of glucose		A maximum of 127 g/L at 36 h		
	CEH: 180 g/L of glucose	T = 303 K, pH of 5.5, 36 h. The agitation rate is set at 220 rpm.	109.5 g/L	Batch fermentation in a shaken flask	
		T = 303 K, pH of 5.5, 18 h. Aeration and agitation rates are set at 3 vvm and 500 rpm.	132.9 g/L	Air-aerated stirred bioreactor (A-ASB)	
		T = 303 K, pH of 5.5, 18 h. Aeration and agitation rates are set at 3 vvm and 500 rpm, the gas inlet pressure is maintained at 0.02–0.05 MPa	181.3 g/L	Supply sealed stirred bioreactor (COS-SSB)	
*Gluconobacter japonicus* CECT 8443	Strawberry purée (50 g/L of glucose + fructose with a ratio of 1:1)	T = 302 K, pH of 3.35–2.9, 20 h. The agitation rate is set at 500 rpm.	0.83 g/g (76%)	Batch fermentation	[93]
*Klebsiella pneumoniae*	Pure glucose (100 g/L)	T = 310 K, pH of 7 (first-stage fermentation), pH of 5 (second-stage fermentation), 12 h. The agitation rate is set at 500 rpm, bottles of the glucose solution (60% in weight) are added when the glucose level in the fermentation broth decreases to about 10–20 g.	1 g/g (422 g/L)	Fed-batch fermentation	[94]
*Penicillium chysogenum*	Pure glucose (50 g/L)	T = 311 K, pH of 5.5, 96 h. The agitation rate is set at 150 rpm.	15.6 g/L	Submerged fermentation	[95]
	Pure glucose (100 g/L)		31.2 g/L		
	Pure glucose (150 g/L)		24 g/L		
	Pure glucose (200 g/L)		20 g/L		
*Penicillium frequentans*	Glucose (120 g/L)	T = 298 K, the initial pH is 6, 7 days, gamma irradiated (at 0.1 kGy), *P. frequentans* incubated at 301 K.	32.13 g/L	Submerged fermentation	[96]
		The same, but T = 303 K.	42.90 g/L		
		The same, but T = 308 K.	27.15 g/L		
		The same, but T = 313 K.	0 g/L		
		T = 303 K, pH of 4, 7 days, gamma irradiated (at 0.1 kGy), *P. frequentans* incubated at 303 K.	26.04 g/L		
		The same, but the initial pH is 5.	38.12 g/L		
		The same, but the initial pH is 6.	44.16 g/L		
		The same, but the initial pH is 7.	28.97 g/L		
		The same, but the initial pH is 8.	20.14 g/L		
	Grape must (120 g/L of glucose)	T = 303 K, pH of 6, 7 days, gamma irradiated (at 0.1 kGy).	44.75 g/L		
	Banana must (120 g/L of glucose)		47.15 g/L		
	Crude molasses (120 g/L of glucose)		51.18 g/L		
*Penicillium puberulum*	Glucose (120 g/L)	T = 298 K, the initial pH is 6, 7 days, gamma irradiated (at 0.1 kGy), *P. frequentans* incubated at 28 °C.	45.06 g/L	Submerged fermentation	[96]
		The same, but T = 303 K.	58.18 g/L		
		The same, but T = 308 K.	31.17 g/L		
		The same, but T = 313 K.	0 g/L		
		T = 303 K, pH of 4, 7 days, gamma irradiated (at 0.1 kGy), *P. frequentans* incubated at 303 K.	32.39 g/L		
		The same, but the initial pH is 5.	40.17 g/L		
		The same, but the initial pH is 6.	58.41 g/L		
		The same, but the initial pH is 7.	42.50 g/L		
		The same, but the initial pH is 8.	30.07 g/L		
	Grape must (120 g/L of glucose)	T = 303 K, pH of 6, 7 days, gamma irradiated (at 0.1 kGy).	52.75 g/L		
	Banana must (120 g/L of glucose)		56.37 g/L		
	Crude molasses (120 g/L of glucose)		63.14 g/L		
*Penicillium oxalicum* 114-2 (CGMCC 5302)	Corn cob residue from xylitol production, 10 g/L of wheat bran, 10 g/L of peptone, 10 g/L of glucose	T is set up to 303 K from 0 to 120 h and then raised up to 318 K after 120 h to 192 h with 20 g/L of the filter paper powder. The agitation rate is set at 200 rpm.	13.54 g/L	Fed-batch, two-stage temperature control strategy	[97]
*Zymomonas mobilis*	Glucose + fructose (400 mmol/L)	T = 312 K, pH of 6.4, 24 h. Aeration and agitation rates are set at 1 vvm and 100 rpm, untreated cells.	0 mmol/L	Batch stirred tank reactor	[98]
		The same, but cells are treated with 0.5% (*v*/*v*) glutaraldehyde.	356 mmol/L (0.94 mmol/mmol)		
	Glucose + fructose (700 mmol/L)	T = 312 K, pH of 6.4, 24 h. Aeration and agitation rates are set at 1 vvm and 100 rpm, untreated cells.	186 mmol/L (0.29 mmol/mmol)		
		The same, but cells are treated with 0.5% (*v*/*v*) glutaraldehyde.	620 mmol/L (0.97 mmol/mmol)		
*Zymomonas mobilis* cells immobilized in glutaraldehyde-cross-linked calcium alginate beads	Glucose + fructose (400 mmol/L)	T = 312 K, pH of 6.4, 24 h. Aeration and agitation rates are set at 1 vvm and 100 rpm.	355 mmol/L (0.94 mmol/mmol)		
	Glucose + fructose (700 mmol/L)	T = 312 K, pH of 6.4, 24 h. Aeration and agitation rates are set at 1 vvm and 100 rpm.	590 mmol/L (0.92 mmol/mmol)		

**Table 3:** Reaction conditions and heterogeneous catalysts used for the glucose oxidation process.

Catalyst	O_2_	T, K	Glucose Concentration	Glu:Me (mol/mol)	t, h	pH	X, %	S, %	Y, %	Ref.
1.8 %Pd/C	0.1 MPa (30 mL/min)	Room	270 mg (1.5 mmol)	100	2	-	100	98	98	[103]
1% Pd/cellulose	30 mL/min	Room	180 mg (1 mmol)	270	3	-	100	91.2	91.2	[104]
0.5% Pt/TiO_2_	0.1 MPa O_2_	318	2.62 g (0.0146 mol)	5700	6	-	13.0	100	13.0	[105]
0.5% Pt/TiO_2_					12		33.1	81.1	26.8	
0.5% Pt/TiO_2_					24		60.9	50.0	30.4	
0.5% Pt-0.5% Cu/TiO_2_					6		100	37.7	37.7	
0.5% Pt-0.5% Cu/TiO_2_					12		100	22.8	22.8	
0.5% Pt-0.5% Cu/TiO_2_					24		100	10.0	10.0	
5% Pd–5% Bi/C	1 L/min	333	1 M (72 g)	no data	2	9	69	100	69	[108]
5% Pd–5% Tl/C							42	50	21	
5% Pd–5% Sn/C							37	28	10	
5% Pd–5% Co/C							21	3	<1	
5% Pd/SiO_2_	0.9 L/min.	333	1 M (45 g)	530	2	9	59.4	73.5	43.7	[109]
5% Pd–0.3% Te/SiO_2_							83.2	91.5	76.1	
5% Pd–0.5% Te/SiO_2_							86.6	91.0	78.8	
5% Pd–1% Te/SiO_2_							100	100	100	
5% Pd–2% Te/SiO_2_							96.2	99.7	95.9	
5% Pd–5% Te/SiO_2_							76.1	97.4	74.1	
5% Pd–8% Te/SiO_2_							48.8	95.6	46.6	
5% Pd/Al_2_O_3_							48.9	87.2	42.6	
5% Pd–1% Te/Al_2_O_3_							100	100	100	
5% Pd–2% Te/Al_2_O_3_							100	100	100	
5% Pd–1% Bi/SiO_2_							63.0	78.0	49.1	
5% Pd–5% Bi/SiO_2_							64.0	89.0	57.0	
5% Pd–8% Bi/SiO_2_							82.0	92.0	75.4	
1.3% Pd/Al_2_O_3_	10 mL/min	333	0.6 M (3.1 g)	5000	2.5	9	29.1	93.1	27.1	[110]
3.5% Pd-2.4% Bi/Al_2_O_3_ (Pd3:Bi1)							56.6	>99.9	56.6	
2.8%Pd-2.3%Bi/Al_2_O_3_ (Pd5:Bi2)							52.2	99.2	51.8	
2.5%Pd-2.3%Bi/Al_2_O_3_ (Pd2:Bi1)							47.5	99.7	47.4	
2.3%Pd-4.3%Bi/Al_2_O_3_ (Pd1:Bi1)							42.1	>99.9	42.1	
1.1%Pd-3.9%Bi/Al_2_O_3_ (Pd1:Bi2)							27.8	>99.9	27.8	
3.5%Pd-2.4%Bi/Al_2_O_3_ (Pd3:Bi1)	10 mL/min	333	0.6 M (3.1 g)	15,000	2.5	9	17.4	92.0	16.0	[111]
				7500			37.6	>99.9	37.6	
				5000			56.6	>99.9	56.6	
				2500			92.8	>99.9	92.8	
				1250			100.0	95.5	95.5	
				500			100.0	86.2	86.2	
	10 mL/min	293	0.6 M (3.1 g)	5000	2.5	9	1.7	>99.9	1.7	
		303					6.9	>99.9	6.9	
		313					17.6	>99.9	17.6	
		323					25.0	>99.9	25.0	
		333					56.6	>99.9	56.6	
		343					47.4	78.0	36.9	
		353					55.2	59.9	33.2	
		363					65.2	25.3	16.5	
	10 mL/min	333	0.6 M (3.1 g)	5000	2.5	6	3.6	>99.9	3.6	
						7	17.1	>99.9	17.1	
						8	51.0	>99.9	51.0	
						9	56.6	>99.9	56.6	
						10	72.9	94.3	68.7	
						11	78.3	80.8	63.3	
						12	88.1	43.2	38.1	
3.94%Au/CeO_2_	0.1 MPa (20 mL/min)	393	0.2 M	100	18	-	74	91	67	[115]
2.36%Au/CeO_2_(20 wt%)/Al_2_O_3_							81	96	78	
2.31%Au/CeO_2_(25 wt%)/ZrO_2_							77	85	65	
2.39%Au/CeO_2_(50 wt%)/ZrO_2_							82	86	71	
1 wt% Au/TiO_2_ (conventional impregnation)	3 bar	433	200 mg	440	1	-	30.3	66.0	20.0	[116]
1 wt% Au/TiO_2_ (modified impregnation)							22.8	23.2	5.3	
1 wt% Au/TiO_2_ (deposition–precipitation)							61.7	94.0	58.0	
1 wt% Au/TiO_2_ (sol-immobilization method)							71.1	94.6	67.3	
Au/CMK-3	0.3 MPa	383	360 mg	1000	2	-	92.4	87.5	80.9	[117]
Au/SBA-15							67.0	92.4	61.9	
Au/CNTs							62.0	82.7	51.3	
Au/graphene							55.6	74.0	41.1	
Au/graphite							54.5	84.1	45.8	
Au/AC							20.8	91.4	19.0	
Au/ZrO_2_							12.7	91.9	11.7	
2%Au/Al_2_O_3_	10 mL/min	333	0.6 M	17,000	7	9	97	96	93	[118]
2%Au/Al_2_O_3_				11,000			100	95	95	
2%Au/Al_2_O_3_				9000			96	97	93	
2.5%Au/Al_2_O_3_				7000			100	97	97	
2.5%Au/Al_2_O_3_				5500			85	93	79	
2%Au/Al_2_O_3_				4000			38	75	28	
2%Au/Al_2_O_3_				3600			70	88	62	
2%Au/Al_2_O_3_				750			27	53	14	
1.1%Au/Al_2_O_3_				6000			94	96	90	
1.4%Au/Al_2_O_3_				6000			97	97	94	
Au/TiO_2_	1 MPa	383	0.3 M (810 mg)	880	4	-	48.1	67.6	32.5	[119]
2%Pt/TiO_2_				890			38.2	63.2	21.1	
2%Au-2%Pt/TiO_2_				880			100.0	57.1	57.1	
2%Pt-2%Co/TiO_2_				890			38.8	48.9	19.0	
2%Pt-2%Mn/TiO_2_				890			37.2	49.8	18.5	
2%Au-2%Co/TiO_2_				880			78.1	62.2	48.6	
2%Au-2%Mn/TiO_2_				880			72.2	41.6	30.0	
0.2%Au/CeO_2_	0.5 MPa	383	2.0 mmol (360 mg)	1000	2	-	25.6	99.4	25.4	[120]
0.2%Au/TiO_2_							11.8	87.9	10.4	
0.2%Au/HAP							47.9	32.0	15.3	
0.2%Au/LDH							75.8	54.0	40.9	
0.2%Au/HAP-LDH							98.9	99.7	98.6	
0.2%Au/(HAP + LDH)							82.4	57.3	47.2	
0.5%Au/HAP-LDH							100	99.3	99.3	
2%Au/HAP-LDH							90.7	94.1	85.3	
0.2%Au/HAP-LDH ^a^							59.6	47.7	28.4	
0.2%Au/HAP-LDH ^b^							84.8	73.7	62.5	

HAP—hydroxyapatite; LDH—Ca–Al layered double hydroxide; HAP-LDH—hybrid composite of HAP and LDH; HAP + LDH—supported Au sample over a physical mixture of HAP and LDH (Ca/P molar ratio = 1.2); ^a^—Ca/P molar ratio = 0.9; ^b^—Ca/P molar ratio = 1.8.

**Table 4:** Reaction conditions and electrocatalysts used for the glucose oxidation process.

Catalyst	Glucose Concentration	T, K	Electrolyte	Process Characteristics	t	pH	X, %	S, %	η_F_, %	Ref.
Au disk	10 mmol	unknown	0.1 M NaOH, 0.1 M NaClO_4_	0.55 V_RHE_, 0 rpm	2 h	-	-	-	22	[129]
				0.55 V_RHE_, 900 rpm			-	-	110	
				0.55 V_RHE_, 2500 rpm			-	-	64	
				0.8 V_RHE_, 0 rpm			-	-	6	
				0.8 V_RHE_, 900 rpm			-	-	110	
				0.8 V_RHE_, 2500 rpm			-	-	23	
				1.1 V_RHE_, 0 rpm			-	-	56	
				1.1 V_RHE_, 900 rpm			-	-	69	
				1.1 V_RHE_, 2500 rpm			-	-	7	
Pd_3_Au_7_/C	0.1 M	293	0.1 M NaOH	0.4 V_RHE_, 50 rpm. Current density—2.58 mA/cm^2^: 2 on Au/C and 0.92 mA/cm^2^ on Pd/C	6 h	-	67	87	65	[130]
Absence of a catalyst	0.04 M	278	0.1 M NaOH	-	65 h	13	18.4	10.7	-	[131]
		293					46.3	4.6	-	
Cu	0.04 M	278	0.1 M NaOH	0.84 V_RHE_	65 h	13	-	30.4	-	
				1.11 V_RHE_			-	44.5	-	
				1.80 V_RHE_			-	17.8	-	
Pt	0.04 M	278	0.1 M NaOH	0.70 V_RHE_ for 30 s, 2.40 V_RHE_ for 1 s, 0 V_RHE_ for 1 s	65 h	13	-	68.0	-	
			0.1 M NaOH	1.10 V_RHE_ for 30 s, 2.40 V_RHE_ for 1 s, 0 V_RHE_ for 1 s			-	78.4	-	
Au	0.04 M	278	0.1 M NaOH	0.55 V_RHE_ for 30 s, 2.40 V_RHE_ for 1 s, 0 V_RHE_ for 1 s	65 h	13	-	86.6	-	
				1.34 V_RHE_ for 30 s, 2.40 V_RHE_ for 1 s, 0 V_RHE_ for 1 s			-	65.8	-	
Ti	50.5 mmol/L	303	10 g/L Na_2_SO_4_	Current density—3 mA/cm^2^, 2.4 V	16 min	7	11	1	-	[100]
0.85%MnO_2_/Ti							55	56	-	
3.06%MnO_2_/Ti							57	39	-	
4.98%MnO_2_/Ti							64	37	-	
5.56%MnO_2_/Ti							45	34	-	
4.98%MnO_2_/Ti	25.3	303	10 g/L Na_2_SO_4_	Current density—3 mA/cm^2^, 2.4 V	19 min	7	99	34	-	
	50.5						92	43	-	
	75.7						80	46	-	
	101						60	47	-	
	126.3						48	51	-	
	50.5	303	10 g/L Na_2_SO_4_	Current density—3 mA/cm^2^, 2.4 V	19 min	2	90	42	-	
						4	91	43	-	
						7	92	45	-	
						8	92	38	-	
						10	93	37	-	
	50.5	288	10 g/L Na_2_SO_4_	Current density—3 mA/cm^2^, 2.4 V	19 min	7	84	20	-	
		303					93	45	-	
		318					83	27	-	
		333					75	24	-	
		348					61	23	-	

**Table 5:** The summary of the glucose electrooxidation to glucaric acid.

Catalyst	Glucose Concentration, mmol	T, K	Electrolyte	Process Characteristics	t	pH	X, %	S, %	η_F_, %	Conversion, % Glucose	Ref.
MnO_2_	25.3	303		Current density—3 mA/cm^−2^	19 min	7.0	-	58	-	99	[100]
	50.5	303		Current density—4 mA/cm^−2^			42	48	-	92	
	75.7	303		Current density—3 mA/cm^−2^			-	42	-	80	
	50.5	288		Current density—3 mA/cm^−2^			-	65	-	84	
	50.5	318		Current density—3 mA/cm^−2^			-	45	-	83	
TEMPO	20	278	Na_2_CO_3_	Current intensity—200 mA	-	12.0	80	-	20	-	[204]
	100 (sodium D-gluconate)	278	Na_2_CO_3_	Current intensity—600 mA	-	12.2	85	-	26	-	
NiFeOx-NF	10	-	0.1 M KOH	Current density—17.7 mA cm^−2^, V_RHE_—1.30 V, TOF—0.03 s^−1^	2 h	13.9	83.3	-	87	98.3	[32]
	50	-		Current density—61.5 mA cm^−2^, V_RHE_—1.30 V, TOF—0.11 s^−1^	10 h	13.9	75.3	-	79	93.1	
	100	-		Current density—87.6 mA cm^−2^, V_RHE_—1.30 V, TOF—0.16 s^−1^	18 h	13.9	71.2	-	73	90.6	
	100	-		Current density—22.1 mA cm^−2^, V_RHE_—1.30 V, TOF—0.04 s^−1^	18 h	13.9	63.8	-	68	92.7	
NiFe(OH)x-NF	100	-		Current density—79.2 mA cm^−2^, V_RHE_—1.30 V, TOF—0.09 s^−1^	18 h	13.9	56.9	-	64	88.6	
Au	100	313	0.1 M Na_2_CO_3_	Two-step synthesis. 1 step: converting D-glucose to D-gluconic acid at 0.6 V_RHE;_	48 h	11.3	-	-	-	17.5	[202]
	40 (gluconic acid)	293	0.025 NaOH	Second oxidation step at 1.1 V_RHE_	24 h	11.3	-	89.5	-	25.0	

η_F_—Faradaic efficiency; V_RHE_—(voltage vs. reversible hydrogen electrode).

## Data Availability

No new data were created or analyzed in this study.
